# Fed-batch like cultivation in a micro-bioreactor: screening conditions relevant for *Escherichia coli* based production processes

**DOI:** 10.1186/s40064-015-1313-z

**Published:** 2015-09-11

**Authors:** Csilla Toeroek, Monika Cserjan-Puschmann, Karl Bayer, Gerald Striedner

**Affiliations:** Austrian Centre of Industrial Biotechnology, Muthgasse 11, Vienna, 1190 Austria; Department of Biotechnology, University of Natural Resources and Life Sciences, Muthgasse 18, 1190 Vienna, Austria

**Keywords:** BioLector, *Escherichia coli*, Fed-batch mode, Microtiter plate, Strain screening

## Abstract

**Objectives:**

Recombinant protein production processes in *Escherichia coli* are usually operated in fed-batch mode; therefore, the elaboration of a fed-batch cultivation protocol in microtiter plates that allows for screening under production like conditions is particularly appealing.

**Results:**

A highly reproducible fed-batch like microtiter plate cultivation protocol for *E. coli* in a micro-bioreactor system with advanced online monitoring capabilities was developed. A synthetic enzymatic glucose release medium was employed to provide carbon limited growth conditions without external substrate feed and the required buffer capacity to keep the pH value within 7 ± 1. Accurate process design allowed for cultivation up to cell densities of 10 g biomass l^−1^ without any limitations in oxygen supply [dissolved oxygen (DO) level above 30 %]. In the micro-bioreactor system (BioLector) online monitoring of cell growth, DO and pH was performed. Furthermore, the influence of the cultivation temperature, the applicability for different host strains as well as the transferability of results to lab-scale bioreactor cultivations was evaluated.

**Conclusion:**

This robust microtiter plate cultivation protocol allows for screening of *E. coli* systems under conditions comparable to lab-scale bioreactor cultivations.

## Background

During high level expression of recombinant proteins in *Escherichia coli* different factors such as the expression system, the localization of the protein, the host strain and the cultivation conditions contribute to protein quality and quantity. Therefore, it is often necessary to initially screen a vast number of clones under many different cultivation conditions. High throughput cultivation systems such as shake flasks, microtiter plates (MTP), small scale stirred tank reactors, and microfluidic devices are used for this initial screening (Bareither and Pollard 2011; Betts and Baganz 2006). The screening cultivations in these small scale systems are usually operated in batch mode where all the media components are present from the start of the cultivation and high substrate concentrations enable fast growth. However, high growth rates often cause oxygen shortage and lead to formation of unwanted, growth-inhibiting metabolites (Büchs 2001; Jeude et al. 2006). In contrast to these screening conditions production is usually conducted in fed-batch mode with carbon limitation for growth rate control. This strategy allows for higher cell densities and product yields and prevents by-product formation (Larsson et al. 1997). The differences between screening and production conditions frequently result in selection of clones that behave poorly under production conditions (Jeude et al. 2006). Consequently conditions in screening approaches should be approximated to production conditions.

The goal of our work was to develop a simple, robust and reproducible protocol for fed-batch cultivation of *E.coli* in MTP. We focused on growth conditions similar to those in a lab-scale bioreactor. Thus, the requirements were carbon-limited growth in a defined synthetic medium to high cell densities and sufficient oxygen supply throughout the cultivation. The BioLector micro-fermentation system providing online access to cell density, pH and pO_2_ represents a good compromise between very complex miniaturized stirred tank reactors and simple titer-plates (Kensy et al. 2009; Samorski et al. 2005). More advanced concepts of this technology with glucose feed and pH control based on microfluidics (Funke et al. 2010a, b) were excluded because of increased complexity, reduced number of cultivation wells and high costs for plates. An alternative to implement fed-batch like cultivation conditions in titer plate format is to use enzyme based glucose release media (Krause et al. 2010; Panula-Perälä et al. 2008). In our approach we selected a synthetic enzymatic glucose release in combination with the BioLector system.

## Methods

### Bacterial strains and plasmid

The cultivation experiments were performed with the recA^−^ K-12 strains *E. coli* HMS174 and *E. coli* RV308, the B strain *E. coli* BL21, and the recombinant host *E. coli* HMS174(DE3)(pET11aGFPmut3.1) (Striedner et al. 2003).

### Media and solutions

For BioLector cultivation experiments the synthetic Feed in Time (FIT) fed-batch medium with glucose and dextran as carbon sources (m2p-labs GmbH, Baesweiler, Germany) (Hemmerich et al. 2011) was directly used or diluted with sterile filtered water to gain 50, 67 and 80 % (v/v) FIT mixtures. The FIT fed-batch medium was supplemented with 1 % (v/v) VitaMix. Immediately prior to inoculation 1 % (v/v) of the glucose releasing enzyme mix (EnzMix) was added. The hydrolytic enzyme (glucoamylase) cleaves off glucose residues from a solubilized glucose polymer. The growth rate of the cells is controlled by the activity of the enzyme and the enzymatic glucose release mimics the substrate feed of lab-scale fermentation processes. All media were tempered to 25 °C.

For high cell density (HCD) cultivation experiments minimal media calculated to produce 80 g cell dry mass (CDM) in the batch phase and 1940 g CDM during feed phase were used. The batch medium was prepared volumetrically; the components were dissolved in 5 L RO-H_2_O. The fed-batch medium was prepared gravimetrically; the final weight was 10.2 kg. All components for the fed-batch medium were weighed in and dissolved in RO-H_2_O separately. All components (obtained from MERCK), were added in relation to the theoretical grams of CDM to be produced: the composition of the batch and the fed-batch medium is as follows: 94.1 mg g^−1^ KH_2_PO_4_, 31.8 mg g^−1^ H_3_PO_4_ (85 %), 41.2 mg g^−1^ C_6_H_5_Na_3_O_7_ * 2 H_2_O, 45.3 mg g^−1^ NH_4_SO_4_, 46.0 mg g^−1^ MgCl_2_ * 2 H_2_O, 20.2 mg g^−1^ CaCl_2_ * 2 H_2_O, 50 μL trace element solution, and 3.3 g g^−1^ C_6_H_12_O_6_ * H_2_O. The trace element solution was prepared in 5 N HCl and included 40 g L^−1^ FeSO_4_∙ * 7H_2_O, 10 g L^−1^ MnSO_4_∙ * H_2_O, 10 g L^−1^ AlCl_3_∙ * 6 H_2_O, 4 g L^−1^ CoCl_2_, 2 g L^−1^ ZnSO_4_∙ * 7H_2_O, 2 g L^−1^ Na_2_MoO_2_∙ * 2 H_2_O, 1 g L^−1^ CuCl_2_∙ * 2 H_2_O, and 0.5 g L^−1^ H_3_BO_3_. To accelerate initial growth of the population, the complex component yeast extract (150 mg g^−1^ calculated CDM) was added to the batch medium. Nitrogen level was maintained by adding 25 % ammonium hydroxide solution (w/w) for pH control. Pre-cultures for inoculation were grown in synthetic media calculated to produce 3 g L^−1^.

### BioLector cultivations

All experiments were performed in the BioLector micro-fermentation system in 48-well Flowerplates^®^ (m2p-labs) equipped with optodes for on-line measurement of dissolved oxygen (DO) and pH value (Funke et al. 2009). Bacterial growth was monitored in the BioLector via a backward scattered light measurement at 620 nm. The biomass concentration (g L^−1^) was calculated from light scatter signals with calibration settings obtained by linear regression analysis. The pH dependent fluorescence signal was excited at 470 nm and monitored at an emission of 525 nm. The DO dependent fluorescence signal was excited at 520 nm and detected at an emission of 600 nm. The signals were converted by the BioLector software (BioLection 2.2.0.3) to DO and pH values using pre-defined calibration settings specific for the Flowerplates^®^. The green fluorescent protein (GFP) expression level was monitored at an excitation of 488 nm and an emission of 520 nm. The signal is given in arbitrary units [A.U] and it was also correlated to the specific amount of recombinant protein in mg GFP g^−1^ CDM with a GFP specific ELISA (Reischer et al. 2004).

The cycle time for all parameters was 15 min. Gas-permeable sealing films (m2p-labs) were used to ensure aseptic conditions and to reduce evaporation. The humidity in the incubation chamber was controlled (% rH ≥85 %) and the shaking frequency was 1400 rpm. The total cultivation volume was 800 or 1000 µL. The initial cell density (OD_ini_) was equivalent to an optical density of OD_600_ = 0.5. For inoculation, a deep-frozen (−80 °C) working cell bank (WCB) (OD_600_ = 2) was thawed and biomass was harvested by centrifugation (7500 rpm, 5 min). Cells were washed with 500 µL of the corresponding medium to remove residual glycerol and centrifuged; then, pellets were re-suspended in the total cultivation medium. All cultivations were prepared in six replicates at 37 °C for 40 h. To ensure comparability of cultivations three wells with dilutions of a turbidity standard (NTU200), two wells with pH buffers (pH 5 and pH 7) and one well with medium for sterility control were used as internal plate standards. The cultivation with recombinant protein synthesis was performed with HMS174(DE3)(pET11aGFPmut3.1) in FIT 67 %. Recombinant gene expression was induced with 1 mM IPTG 10 h after start of cultivation.

### HCD cultivations

All HCD fermentations were performed in a 30 L (23 L net volume, 5 L batch volume) computer controlled bioreactor (Bioengineering; Wald, Switzerland) equipped with standard control units (Siemens PS7, Intellution iFIX). The pH was maintained at a set-point of 7.0 ± 0.05 by addition of 25 % ammonia solution (w/w), the temperature was set to 30 ± 0.5 °C. To avoid oxygen limitation the DO level was held above 30 % saturation by adjusting the stirrer speed and the aeration rate of the process air. The maximum overpressure in the head space was 1.0 bar. Foaming was suppressed by addition of 0.5 mL L^−1^ antifoam (PPG 2000 Sigma Aldrich) to the batch medium and by pulsed addition of antifoam during the fed-batch phase. The cultivation was inoculated with a pre-culture. The pre-culture was set-up by inoculating 300 mL LB media with 1 mL of a deep frozen WCB. Cells were grown on an orbital shaker at 200 rpm and at 37 °C until the OD_600_ reached a value of 3.5. Thereafter, approximately 15 mL of the pre-culture, corresponding to 50 OD units, were aseptically transferred to the bioreactor. At the end of the batch phase as soon as cells entered the stationary growth phase, an exponential substrate feed was started. During a period of 11 h cells grew exponentially at a constant growth rate of µ = 0.17 h^−1^. The substrate feed was controlled by increasing pump speed according to the exponential growth algorithm, ${\text{X}} = {\text{X}}_{0} \cdot {\text{ e}}^{{\upmu {\text{t}}}}$, with superimposed feedback control of weight loss in the substrate tank.

Thereafter, the feed regime was switched from exponential to linear feed mode. During the linear feed phase (17 h) glucose was fed to the culture with a constant rate of 4.9 g glucose min^−1^. Due to the linear feeding profile the growth rate decreased from 0.17 h^−1^ to 0.04 h^−1^.

### Calibration of light scatter signals

The correlation between the light scatter signal and the biomass concentration was determined by measuring the scattered light intensities of a concentrated cell suspension and a series of cell suspensions/water dilutions (1:0.3, 1:1, 1:4, 1:9, and 1:19). The cell suspension of an *E.coli* HMS174 shake flask culture (24 h incubation at 37 °C in FIT 67 %) was used for measurement of the light scatter signal in the BioLector at 30 °C and 1400 rpm until a stable initial value was reached. In parallel, the biomass concentration of the culture was determined by centrifugation of 20 mL of the cell suspension, followed by re-suspension of the pellet in distilled water, centrifugation, and transfer to a pre-weighed beaker. The beaker was dried for 24 h at 105 °C and re-weighed.

### Glucose release kinetics

The glucose release was monitored in diluted FIT 67 % at the default pH of 7.3 or after changing the pH to 6.8 with HCl. Shake flasks (250 mL) with a filling volume of 30 mL were incubated for 30 h on an Infors HT Multitron orbital shaker. The temperature was set to 25, 30, or 37 °C and the shaking frequency was 200 rpm. The glucose concentration was determined periodically with an enzymatic assay specific for d-glucose (Megazyme International Ireland Ltd.). Prior to the measurement, the glucose releasing enzyme was inactivated for 15 min at 85 °C and samples were diluted to 0.05–5.00 mg glucose L^−1^.

## Results

In this study we evaluated the suitability of a medium based on enzymatic glucose release (FIT fed-batch medium) for fed-batch like cultivation of *E. coli* in MTP. The predefined protocol must allow for cultivation at a DO level above 30 % and at pH values within 7 ± 1 throughout the process and carbon limited growth conditions in the “feed”-phase.

**Fig. 1:**
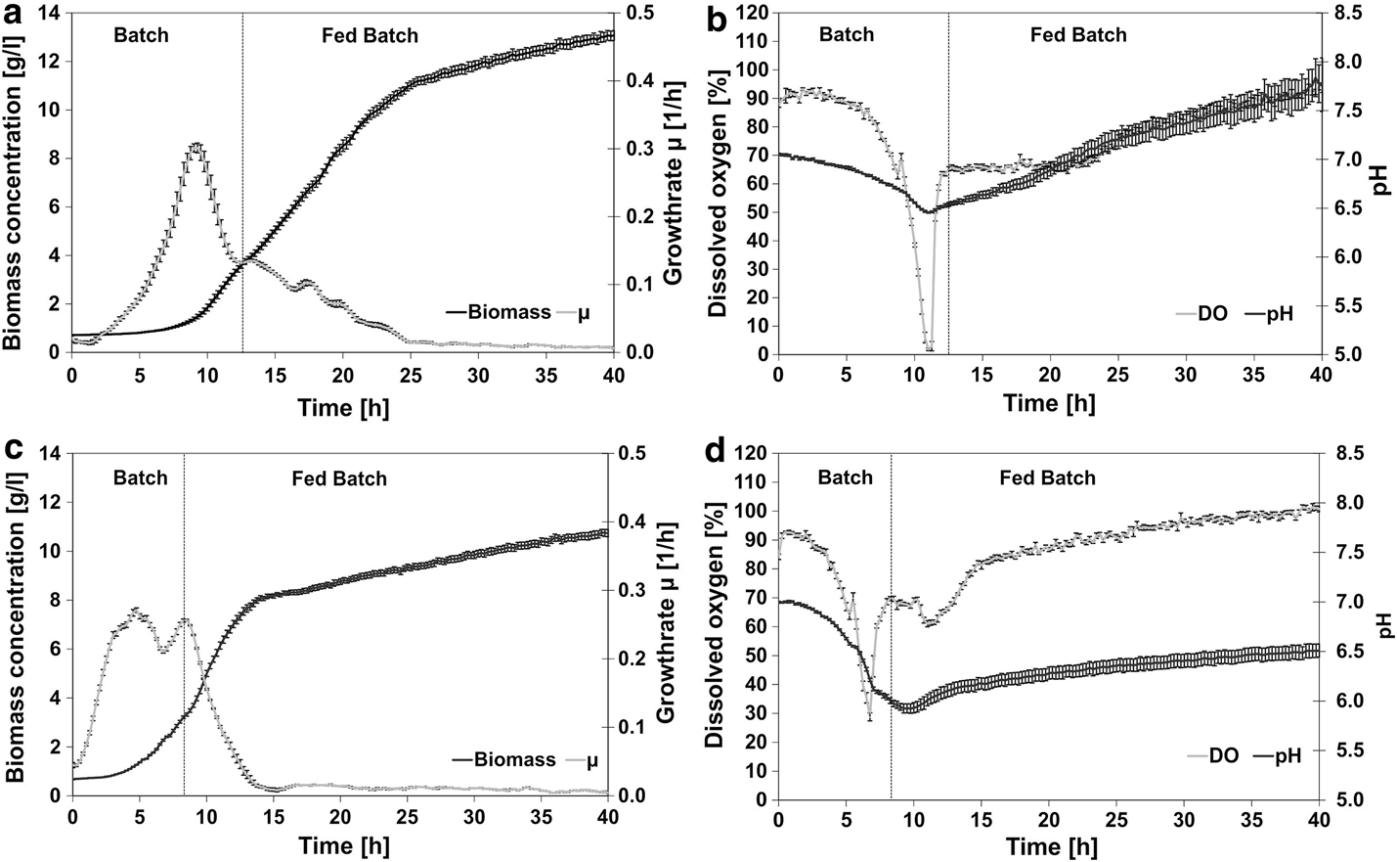
BioLector cultivations of *E.* *coli* HMS174 in enzymatic glucose release media. Biomass course, growth rate (µ), dissolved oxygen (DO) and pH value were analyzed. **a**, **b** Cultivation with FIT 100 % medium and **c**, **d** cultivation with FIT 67 % medium. The mean values and the standard errors of the mean for six individual parallel experiments are shown

In a first setting *E. coli* HMS174 was cultivated in the BioLector in 1000 µL FIT 100 % medium. In these experiments high biomass concentrations of 13 g L^−1^ were reached, but during a long lag phase in the beginning of the cultivation glucose accumulated and was then metabolized at a high rate in the late batch phase. This caused the DO level to drop close to zero (Fig. 1a, b).

Next, the cultivation volume was reduced to 800 µl to increase the oxygen transfer rate (OTR). In parallel diluted FIT fed-batch media (FIT 50 %, FIT 67 %, FIT 80 %) were used to prevent exceeding oxygen consumption rates in the late stage of the batch phase. With FIT 50 % medium a biomass concentration of 12 g L^−1^ was obtained and the batch phase was shortened by 2.5 h compared to cultivation in undiluted medium and there was no oxygen limitation at the end of the batch phase. However, after 10 h, the pH value reached a minimum of 5.5 and for 7 h no growth was detected. Within this resting phase the pH increased to 6.0 and growth was restored. In FIT 80 % medium the batch phase was shortened by 2.5 h and the pH value ranged between 6.3 and 7.0 throughout the cultivation. The DO-level dropped to 5 % at the end of the batch phase. The cultivation with FIT 67 % medium (Fig. 1c, d) reached a final biomass of 11 g L^−1^ in a shortened batch phase without oxygen limitation or severe pH variations.

**Fig. 2:**
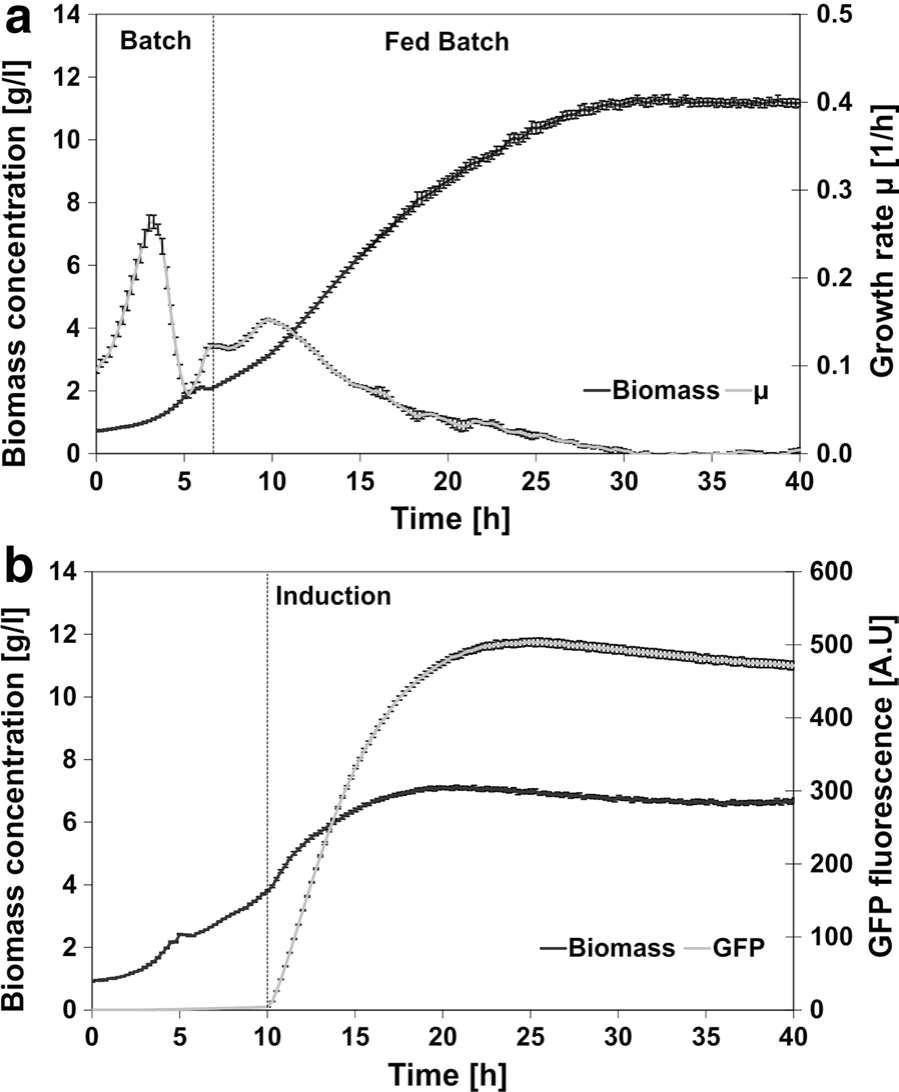
BioLector cultivations of *E.* *coli* HMS174(DE3)(pET11aGFPmut3.1) in FIT 67 % medium. **a** Biomass course and growth rate (µ) of non-induced cells and **b** biomass and GFP signal (given in arbitrary units [A.U]) in a culture with 1 mM IPTG induction. The mean values and the standard errors of the mean for six individual parallel experiments are shown

Based on these results, the recombinant strain HMS174(DE3)(pET11aGFPmut3.1) was cultivated according to the protocol with FIT 67 % with and without induction of GFP expression to evaluate the influence of recombinant protein synthesis on cell growth. The growth behavior of the wild type and the non-induced recombinant strain did not show significant differences (Figs. 1c, 2a). However, overexpression of recombinant protein negatively affected cell growth and the biomass yield was 36 % lower than in the reference experiment without induction (Fig. 2a, b). Ten hours after induction the GFP signal reached a maximum of 500 A.U, which correlates with 90 mg GFP g^−1^ biomass (Fig. 2b).

**Fig. 3:**
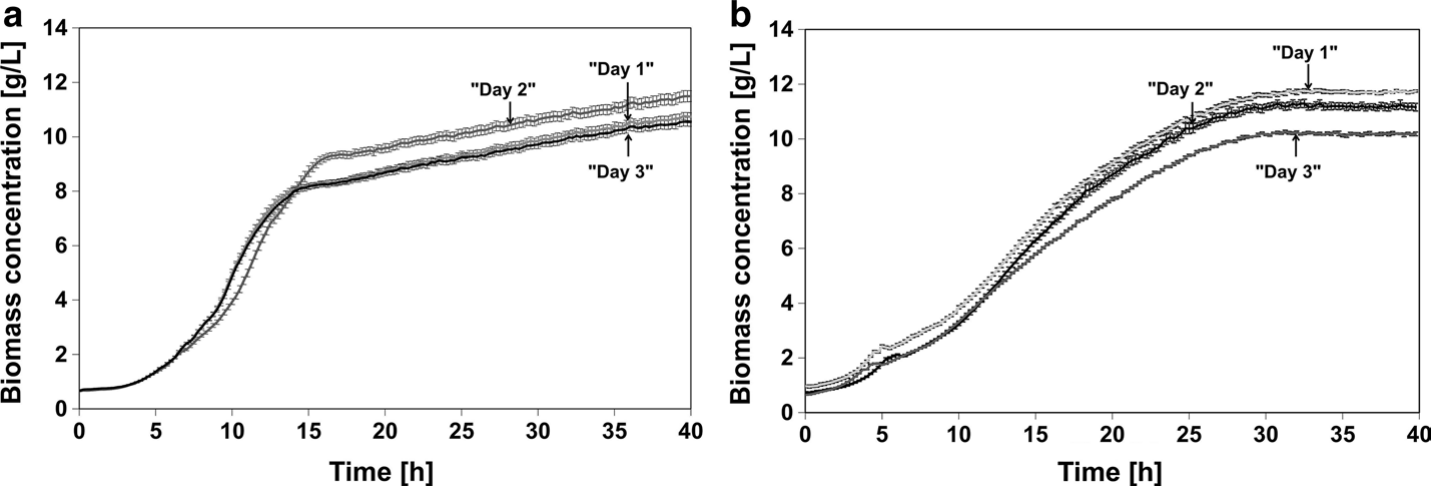
Reproducibility of the established small scale fed-batch cultivations: **a** growth of *E. coli* HMS174 and **b** of *E. coli* HMS174(pET11aGFPmut3.1) in m2p 67 %; “Day 1”, “Day 2” and “Day 3” are biological replicates of a cultivation performed as sixfold; the mean value and the standard error of the mean for these six individual parallel experiments is shown

To evaluate the stability of the µ-scale cultivation protocol, a reproducibility study was conducted. Wild type and recombinant strains were cultivated in sixfold on 3 different days (Fig. 3). For biomass the well-to-well coefficient of variation (CV) on the same plate was 4.5 % for both strains. The day-to-day and plate-to-plate CV was 4.8 % for the wild type strain HMS174 and 9.1 % for HMS174(DE3)(pET11aGFP).

**Fig. 4:**
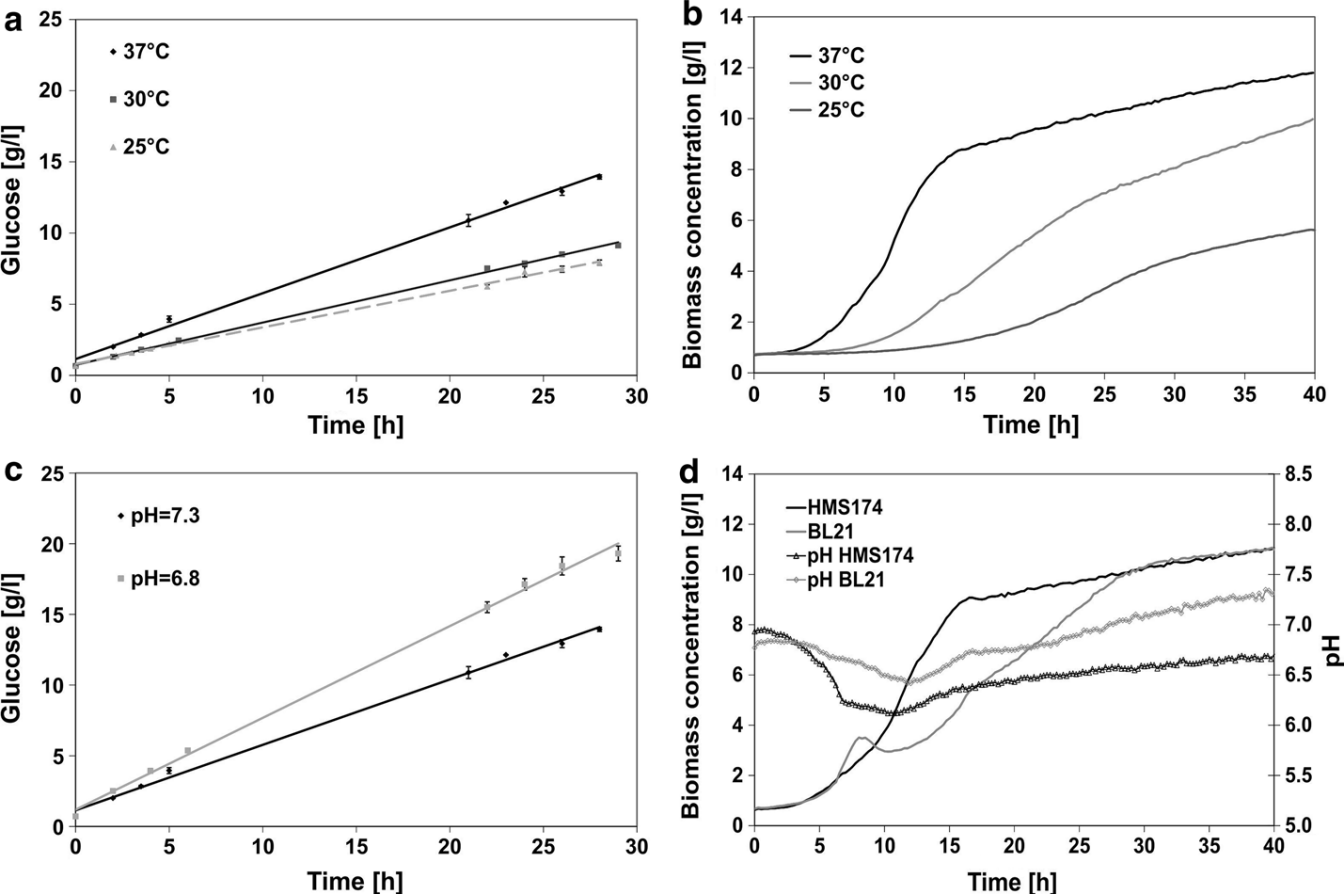
**a**, **c** Influence of temperature and pH on glucose release in m2p 67 % medium; **b** corresponding growth of *E. coli* HMS174 at different temperatures and **d** of *E coli* HMS174 and *E coli* BL21 at 37 °C in m2p 67 %. For glucose release and process characteristics, the mean value for triplicates is shown

To investigate the influence of temperature and pH on the activity of the polymer degrading enzyme the glucose release was determined in in vitro experiments at 25, 30, and 37 °C and pH values of 7.3 and 6.8. The enzyme activity directly correlated with temperature as the glucose concentration after 28 h incubation at 37 °C was 76 % higher than at 25 °C (Fig. 4a). With respect to the variation of pH, incubation at a lower pH of 6.8 yielded 40 % higher glucose concentrations than at pH 7.3 (Fig. 4c).

The strong relationship between temperature and cell growth was shown in cultivation experiments with HMS174 (Fig. 4b). Furthermore, the growth behavior of two strains with different acidification patterns (HMS174 and BL21) was compared (Fig. 4d). During cultivation of HMS174, the pH reached a minimum of 6.1 after 10.5 h; whereas, a minimum of 6.4 was reached after 12 h cultivation of BL21 cells. Within the first 25 h of cultivation, higher growth rates were observed for strain HMS174. Thereafter, the growth of both strains was comparable reaching the same biomass concentration of 11 g L^−1^ after 40 h (Fig. 4d).

**Fig. 5:**
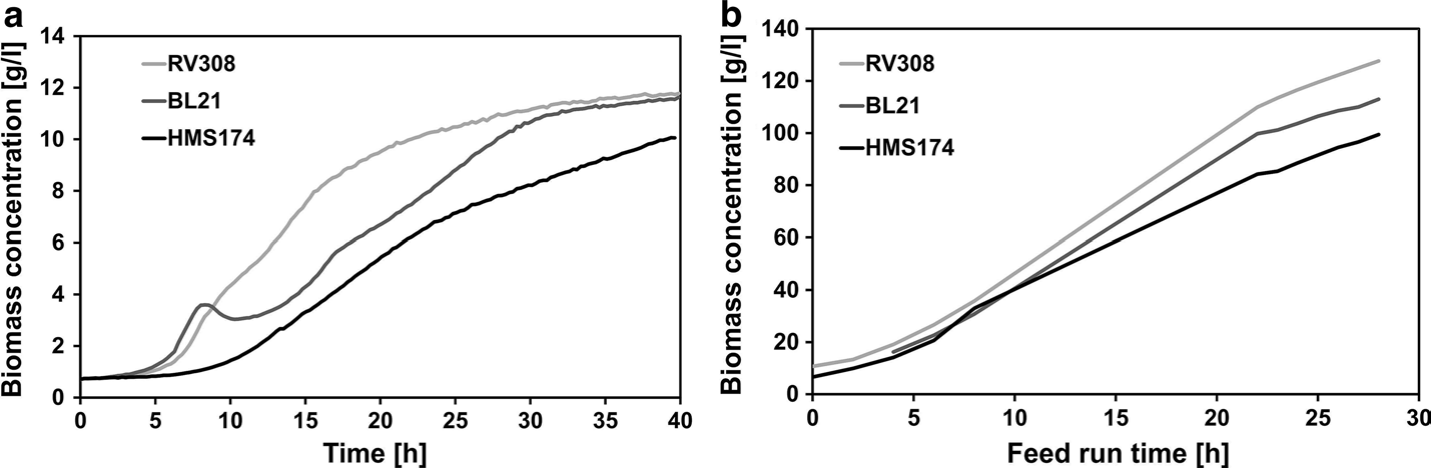
Growth of different *E. coli* strains in micro-bioreactor (**a**) and HCD lab-scale bioreactor (**b**) cultivations. The fed-batch cultivations in both scales were performed at 30 °C according to the standard protocols

To evaluate transferability of results generated in our small scale screening platform to fermentation processes a series of HCD cultivation was conducted with *E. coli* HMS 174, *E. coli* RV308 and *E. coli* BL21 (Fig. 5).

## Discussion

In this work we successfully established a MTP cultivation protocol that allows for *E. coli* clone screening and characterization under production like conditions. The application of a synthetic glucose release medium enabled a fed-batch like *E. coli* cultivation without external substrate feed up to >10 g CDM L^−1^. The predefined success criteria (DO >30 %, pH = 7 ± 1; carbon limited growth in the feed phase) were set in order to mimic production conditions. A similar concept has already been used for *Pichia pastoris* (Wenk et al. 2012) but for *E.coli* reported results were limited to shake flask experiments (Hemmerich et al. 2011). Based on our results we conclude that the FIT 100 % medium supplemented with the enzyme for glucose release cannot directly be used in our approach as the DO level dropped close to zero at the end of the batch phase. Even though the oxygen depletion had no influence on further growth or the final biomass, anaerobic conditions are not favorable, especially during a strain screening approach (Büchs 2001; Zimmermann et al. 2006). To avoid the oxygen limitation at the end of the batch phase the cultivation volume was reduced as lower volumes allow for increased oxygen transfer in titerplates (Funke et al. 2009). Furthermore the use of diluted medium (FIT 67 %) with lower glucose concentration and lower osmotic pressure led to a shortened lag-phase. This combination met all of the defined success criteria important to mimic production conditions and the final cell density at the end of the process was only 8 % lower as in cultivations with FIT 100 %.

The transition from batch to fed-batch mode was clearly shown in experiments with different strains (HMS174, HMS174(DE3)(pET11aGFPmut3.1), BL21) (Figs. 1, 2a). The cells switched from unlimited to carbon limited growth controlled by the constant glucose release rate of the hydrolyzing enzyme. The continuously decreasing growth rate during this carbon limited phase most likely equates the output of a linear feed in lab-scale bioreactors. A high reproducibility was proven for the established cultivation protocol with the FIT 67 % medium and consequently an important requirement for a HTP cultivation platform for strain screening was fulfilled.

As expected the HMS174 wild type strain reached a higher final cell density than its recombinant descendant and the variations in pH and DO were more pronounced but they were kept within defined ranges. In cultivations with induced HMS174(DE3)(pET11aGFPmut3.1) product formation kinetics and GFP yield (90 mg GFP g^−1^ biomass) correspond well to results achieved in fully controlled fed-batch cultivation (129 mg GFP g^−1^ biomass) (Striedner et al. 2010). Moreover, the influence of the high level recombinant gene expression on the final biomass yield with 30 % decrease in fermenter and 36 % decrease in BioLector was comparable in both systems (Fig. 2).

The cultivation temperature represents an important factor in *E. coli* screening approaches as there is influence on growth rate, product formation kinetics and protein folding. The temperature optimum of many microbial glucoamylases ranges from 40 to 60 °C (Kumar and Satyanarayana 2009). In vitro experiments at temperatures relevant for *E. coli* cultivations (25, 30, 37 °C) showed that there is still significant impact on the activity of the hydrolyzing enzyme (Fig. 4a). Consequently the influence of temperature on growth characteristics is more pronounced in enzyme based glucose release media (Fig. 4b). The pH value is another process variable which influences the enzyme activity and the accepted pH range of 7 ± 1 in our setup is higher than the optimum for glucoamylases (Kumar and Satyanarayana 2009). Consequently a decrease from pH 7 to pH 6.8 in in vitro experiments resulted in a significant increase in enzyme activity (Fig. 4c). This phenomenon must be considered in screening approaches with glucose release medium as the amount of metabolically produced acid represents a strain specific attribute (Shiloach et al. 1996). To depict this issue *E. coli* HMS and *E. coli* BL21 were selected as strains with significantly different acetate formation kinetics under feast glucose conditions (Fig. 4d).

Transferability of results to lab scale bioreactor cultivations, a central criterion for a small scale screening protocol, was successfully shown in HCD cultivations. Growth behavior of *E. coli* HMS, *E. coli* RV308 and *E. coli* BL21, relevant production strains with significantly different growth kinetics and acidification patterns, were compared in both scales. With respect to glucose yield coefficient and growth we ended up with the same ranking (Fig. 5).

## Conclusions

This robust MTP cultivation protocol allows for screening of *E. coli* systems under conditions comparable to lab-scale bioreactor cultivations. Small scale cultivations characterized by carbon limited growth, high final cell densities, and no limitations in oxygen supply generate results directly transferable to larger scales and HCD cultivation. Concerning throughput the protocol represents a good compromise suited for medium size screening approaches in early process development already focused on production processes. The next step will be the evaluation whether the established protocol can also be used for identification of optimal cultivation and production conditions transferable to larger scales.

This robust high throughput protocol significantly improves and accelerates the screening procedures for *Escherichia coli* and facilitates cost reduction in early process development.
